# An investigation of machine learning methods in delta-radiomics feature analysis

**DOI:** 10.1371/journal.pone.0226348

**Published:** 2019-12-13

**Authors:** Yushi Chang, Kyle Lafata, Wenzheng Sun, Chunhao Wang, Zheng Chang, John P. Kirkpatrick, Fang-Fang Yin

**Affiliations:** 1 Medical Physics Graduate Program, Duke University, Durham, North Carolina, United States of America; 2 Department of Radiation Oncology, Duke University Medical Center, Durham, North Carolina, United States of America; 3 School of Information Science and Engineering, Shandong University, Qingdao, Shandong, Shandong, People’s Republic of China; 4 Duke Kunshan University, Kunshan, People’s Republic of China; University of Pennsylvania, UNITED STATES

## Abstract

**Purpose:**

This study aimed to investigate the effectiveness of using delta-radiomics to predict overall survival (OS) for patients with recurrent malignant gliomas treated by concurrent stereotactic radiosurgery and bevacizumab, and to investigate the effectiveness of machine learning methods for delta-radiomics feature selection and building classification models.

**Methods:**

The pre-treatment, one-week post-treatment, and two-month post-treatment T1 and T2 fluid-attenuated inversion recovery (FLAIR) MRI were acquired. 61 radiomic features (intensity histogram-based, morphological, and texture features) were extracted from the gross tumor volume in each image. Delta-radiomics were calculated between the pre-treatment and post-treatment features. Univariate Cox regression and 3 multivariate machine learning methods (L1-regularized logistic regression [L1-LR], random forest [RF] or neural networks [NN]) were used to select a reduced number of features, and 7 machine learning methods (L1-LR, L2-LR, RF, NN, kernel support vector machine [KSVM], linear support vector machine [LSVM], or naïve bayes [NB]) was used to build classification models for predicting OS. The performances of the total 21 model combinations built based on single-time-point radiomics (pre-treatment, one-week post-treatment, and two-month post-treatment) and delta-radiomics were evaluated by the area under the receiver operating characteristic curve (AUC).

**Results:**

For a small cohort of 12 patients, delta-radiomics resulted in significantly higher AUC than pre-treatment radiomics (p-value<0.01). One-week/two-month delta-features resulted in significantly higher AUC (p-value<0.01) than the one-week/two-month post-treatment features, respectively. 18/21 model combinations were with higher AUC from one-week delta-features than two-month delta-features. With one-week delta-features, RF feature selector + KSVM classifier and RF feature selector + NN classifier showed the highest AUC of 0.889.

**Conclusions:**

The results indicated that delta-features could potentially provide better treatment assessment than single-time-point features. The treatment assessment is substantially affected by the time point for computing the delta-features and the combination of machine learning methods for feature selection and classification.

## I. Introduction

Radiomics is being actively investigated by computing high-dimensional quantitative features from medical images (CT, MRI, PET, etc.) to provide predictive, prognostic, or diagnostic decision support[1, 2]. As radiomics is based on inherent information encoded in standard-of-care images, it could potentially become a noninvasive, low-cost, and patient-specific decision support tool for routine clinical practice. Due to the high dimensionality and complexity of radiomics data, machine learning has been a critical component for feature analysis in radiomics studies. Notable examples include predicting overall survival[3, 4], tumor staging[5], tumor histology classification[6–8], recurrence[9, 10] and other clinical endpoints for lung[3, 7], breast[9], head and neck[4], prostate[11], pulmonary nodule[12], thyroid[13, 14], bladder^21^, and brain[15] tumors.

Features in radiomic studies are typically defined in one of two ways: (1) single-time-point radiomics, where features are extracted from a particular image (e.g., pre-treatment)[6, 16, 17], and (2) delta-radiomics, where features are extracted from a time series of images (e.g., pre- and post-treatment)[18]. The latter technique reflects the temporal change of radiomic features. In particular, delta-radiomics have been shown to be effective in assessing the response of colorectal liver metastases to chemotherapy[19], differentiating radiation pneumonitis following radiotherapy (RT)[20], predicting overall survival (OS) of patients with non-small cell lung cancer (NSCLC) treated with RT[21], and predicting OS, progression-free survival, and early/late progressors for recurrent glioblastoma multiforme to bevacizumab treatment[22].

While delta-radiomics has therefore demonstrated promising results, there are only a few studies that have compared its effectiveness to that of single-time-point radiomics. In particular, Zhang et. al.[23] extracted features from two follow-up MR images for brain metastases after gamma knife radiosurgery to differentiate radiation necrosis from tumor progression. They compared the delta-radiomics to single-time-point radiomics from the second follow-up image, and reported the prediction accuracy of the former was slightly higher (73.2%) than that of the latter (69.1%). Further, Fave et. al.[18] used clinical factors, pre-treatment features, and delta-features to assess the response of NSCLC to RT and concurrent chemotherapy. They reported that only one delta-feature, texture strength, was prognostic in predicting local-regional recurrence, and that no delta-features were more prognostic in predicting OS or distant metastases than clinical factors and pre-treatment features. Further investigation of delta-radiomics is warranted to better understand its potential advantages.

In previous studies that consider delta-radiomics, while statistical associations have been studied, machine learning has not been widely used for feature analysis. For example, in many delta-radiomics cases, statistical methods like Spearsman’s correlation and multivariate Cox regression[18, 22, 24] or at most a single machine learning method (logistic regression) have been performed[19–21]. One study[23] used 5 different machine learning methods, but only for building classification models, and did not study machine learning based feature selection techniques. Several single-time-point radiomics studies [3, 15, 25] have demonstrated a substantial impact of machine learning technique on both feature selection and classification. Therefore, a systematic study regarding this impact on both feature selection and classification in radiomics/delta-radiomics analysis is highly desirable.

This study aims to investigate the effectiveness of delta-radiomics compared to single-time-point radiomics in predicting OS following concurrent brain radiosurgery and bevacizumab treatment. Radiomic features were extracted from the gross tumor volume (GTV) of both T1-weighted and T2-weighted FLAIR MR images acquired at the following time points: (1) pre-treatment, (2) one-week post-treatment, and (3) two-month post-treatment. We used a univariate Cox regression model and 3 machine learning methods for feature selection, as well as 7 machine learning classification models. ROC analysis was used to compare the predictive performance of different combinations of feature selection and classification models for 3 categories of single-time-point radiomics (pre-treatment, one-week post-treatment, and two-month post-treatment) and 2 categories of delta-radiomics (one-week delta-radiomics, and two-month delta-radiomics).

## II. Materials and methods

### II.A. Materials

In this study, we re-analyzed a specific dataset from a previous study approved by Duke University Health System Institutional Review Board (IRB).[26] All data were fully anonymized before we accessed them. Twelve patients with WHO grade III or IV recurrent malignant gliomas treated by concurrent stereotactic radiosurgery (SRS) and bevacizumab were included. For each patient, we analyzed information of 3D GTV from the pre-treatment, one-week post-treatment (post1-treat), and two-month post-treatment (post2-treat) T1-weighted and T2-weighted FLAIR (Fluid Attenuated Inversion Recovery) MR images acquired with a 1.5T birdcage quadrature head coil MR unit (GE Medical Systems).

The OS data of this cohort was between 5.3 months and 29.4 months, with 6 patients demonstrating an OS shorter than 1 year, and the others longer than 1 year. For feature analysis, OS was assigned as the prediction endpoint and was dichotomized: OS<1 year was labeled as class 0, and OS ≥ 1 year was labeled as class 1.

### II.B. Methods

**Fig 1:**
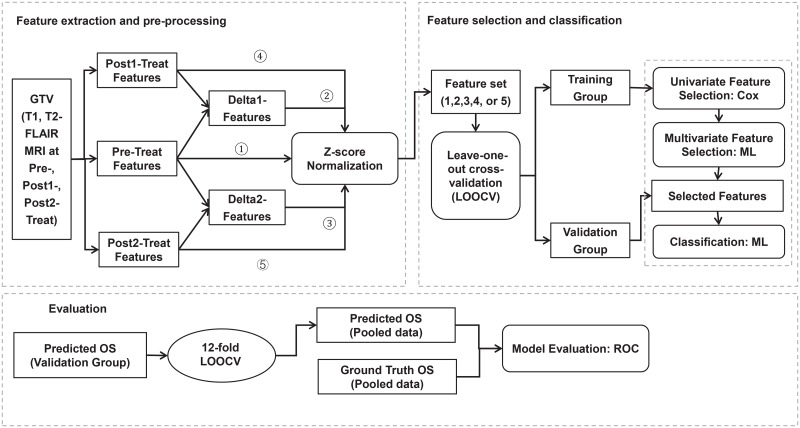
Overall research workflow.

The overall research workflow of this study is shown in Fig 1.

As shown in Fig 1, the GTV was first contoured by an experienced radiation oncologist on the pre-treatment MR and CT images using the Eclipse treatment planning system (Varian Medical Systems, Palo Alto). The post1 and post2 MR images were registered to the pre-treatment MR images in Velocity software (Varian Medical System) using rigid body registration, and the GTV contours delineated were transferred to the post-treatment MR images. The registration was initially performed using an automatic registration algorithm and then manually validated by visually check. The gray scale information content of the MR images was re-binned into 64 grey level shades.

Then, 61 radiomic features (as shown in S1 Appendix) were extracted from the GTV in each MR image, including 22 texture features from the grey-level co-occurrence matrix (GLCOM)[27], 11 texture features from the grey-level run-length matrix (GLRLM)[28], 13 texture features from the grey-level size-zone matrix (GLSZM)[29], 5 features from the neighboring grey-level difference matrix (NGLDM)[30], 6 morphological features, and 4 intensity histogram-based features. The features were extracted by an in-house feature extraction tool developed based on MATLAB 2017a (MATLAB Co. Ltd) using standard mathematical formulae[31]. The delta-features were then computed as the relative changes of post-treatment features to pre-treatment features:

$$\left[\Delta F_{1}=\left(F_{post1}-F_{pre}\right)/F_{pre}\right]$$ (1)

$$\left[\Delta F_{2}=\left(F_{post2}-F_{pre}\right)/F_{pre}\right]$$ (2)

where, *F*_*pre*_ refers to the features extracted from pre-treatment MR images, *F*_*post*1_ and *F*_*post*2_ refer to features computed from post1-treat MR images and post2-treat MR images, respectively. Δ*F*_1_ and Δ*F*_2_ refer to delta-features computed between *F*_*post*1_ or *F*_*post*2_ and *F*_*pre*_, respectively. To ensure normalized units, each of the 5 feature categories (*F*_*pre*_, *F*_*post*1_, *F*_*post*2_, Δ*F*_1_, and Δ*F*_2_) was converted to a z-score distribution prior to feature analyses.

**Fig 2:**
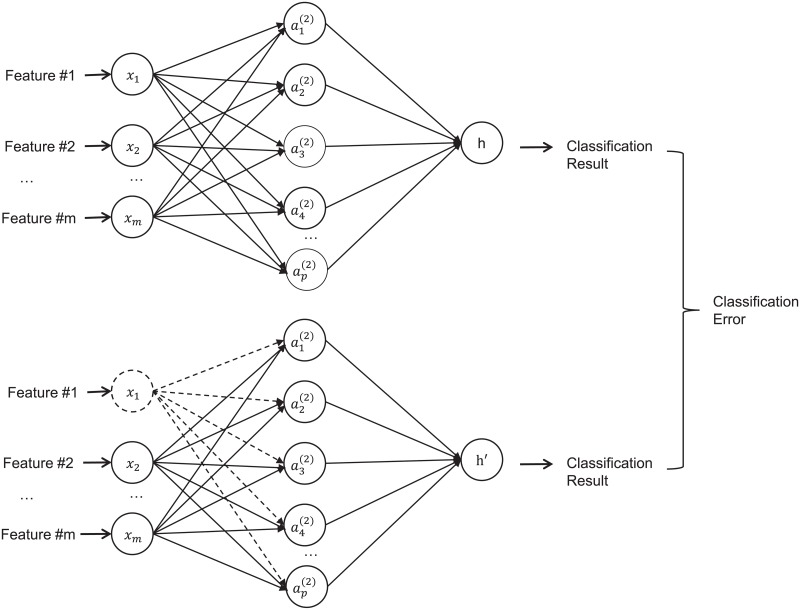
A diagram illustration the neural network structure for feature selection.

Next, each of the 5 feature categories (*F*_*pre*_, *F*_*post*1_, *F*_*post*2_, Δ*F*_1_, and Δ*F*_2_) was separately analyzed for building feature selection and classification models. Leave-one-out cross validation (LOOCV) was adopted to reduce overfitting for this small cohort of patients. The features were sequentially selected using a two-step process: (1) First, a univariate Cox regression model[32] was used, which evaluated whether a model built on this single feature was a better fit than the null model [18]. Only significant features in the univariate Cox regression model (p-value<0.1) were selected. (2) Second, one of the following multivariate machine learning models was used: out-of-bag permutation random forest (RF), neural networks (NN), or L1-regularized logistic regression (L1-LR). The features were selected according to their importance in deciding the OS in each algorithm. In the out-of-bag permutation random RF algorithm[33], the importance of each feature was indicated by the out-of-bag loss. In the NN method, a three-layer back propagation NN was built and the input features were disabled one-by-one for feature selection. “Disable” here means that all weights related to this feature were set to 0, as shown in Fig 2. The importance of the feature was indicated by the classification error when this feature was disabled. In the L1-LR method, the importance of each feature was indicated by the absolute value of the weights in the model. In this experiment, we limited each machine learning feature selection model to select N features. The number N was varied from 1 to 3 in order to choose the optimal parameter for building classification models.

The selected features were then used to build binary classification models using seven commonly used machine learning algorithms: (1) random forest (RF) classifier, built by the “fitcensemble” function in MATLAB 2017a (MathWorks, Natick, MA, USA) with 100 decision trees; (2) L1-regularized logistic regression (L1-LR) classifier, with a sigmoid function as the active function; (3) L2-regularized logistic regression (L2-LR), also with a sigmoid function as the active function; (4) Linear support vector machine (LSVM) classifier, built by the “fitcsvm” function in MATLAB 2017a; (5) Kernel support vector machine (KSVM) classifier, with Gaussian radial basis function as the kernel function; (6) Three-layer back-propagation neural network classifier (NN) [34], with 10 hidden units in the hidden layer; and (7) Naïve bayes classifier (NB) which is based on Bayes’s rule with a strong assumption that features are independent of each other within each class. Although this assumption is hard to be satisfied, naïve bayes appears to work well in practice even when that independence assumption is not valid[35, 36]. These classification models are the commonly used techniques used in previous radiomics studies. [3, 25] The output of each classification model was the score of the predicted class label being equal to 1. Here, score refers to the posterior probability, which describes the probability of predicted class being 1 with the given feature values.

The performance of the models was evaluated via ROC analysis based on the area under the curve (AUC), which is one of the most prevalent evaluation criteria in radiomics studies. Once the feature selection and classification models were built with the training group, the validation group was used to test the models. The scores of the validation groups, after LOOCV, were pooled and then dichotomized by a given decision threshold. The true positive fraction (TPF) and false positive fraction (FPF) were calculated based on the threshold. Then, the decision thresholds varied to obtain all distinct TPFs and FPFs. In practice, the thresholds were selected as all distinct scores. Finally, the AUC was calculated by trapezoidal approximation with all distinct TPFs and FPFs.

Each feature selection and classification model combination was trained and tested for 50 iterations to avoid the random effect. We compared the average AUC of each model combination among *F*_*pre*_, *F*_*post*1_, *F*_*post*2_, Δ*F*_1_, and Δ*F*_2_ by paired-sample t-test.

## III. Results

**Fig 3:**
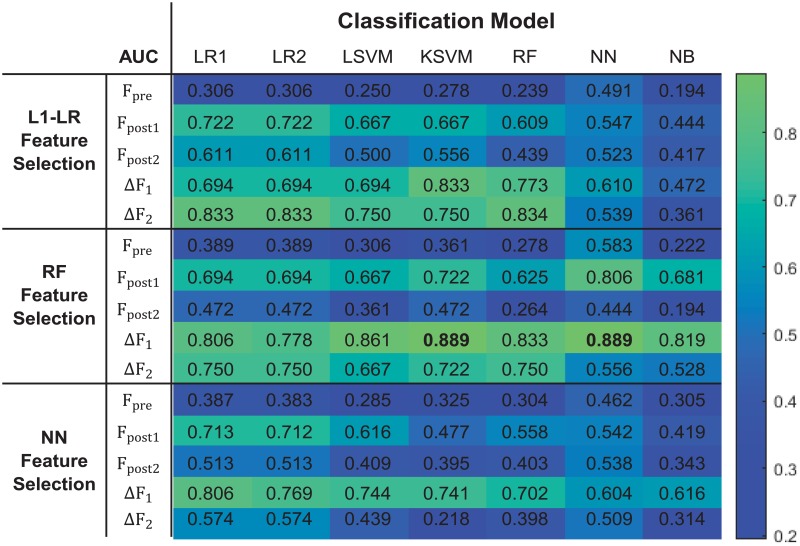
AUC values for all tested feature selection model and classification model combinations.

**Fig 4:**
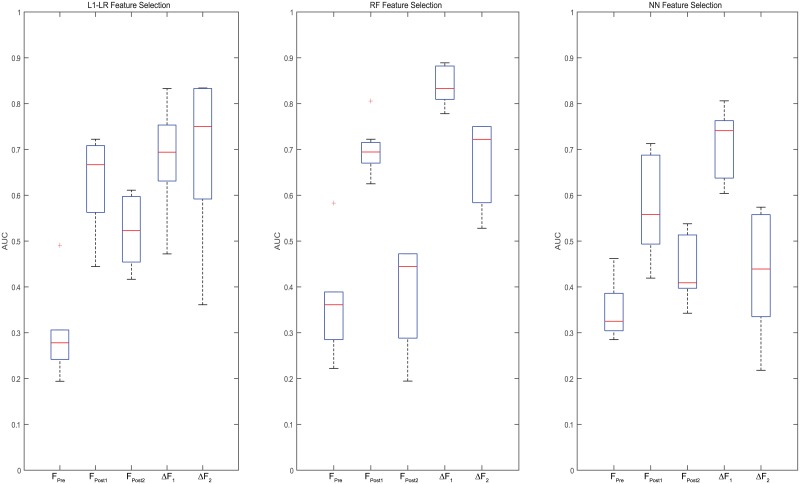
A box plot to display the AUC value range for each feature selection model with seven classification models for each feature category.

The AUC values derived from different combinations of feature selection and classification models are shown in Fig 3. Box plot was used for better illustration of the results in Fig 4, where each box indicates the median and range of AUC values resulted from combinations of a feature selection model with all 7 classification models based on a certain feature category.

Based on paired-sample t-test, models built with Δ*F*_1_ or Δ*F*_2_ showed significantly higher AUCs than those built with *F*_*pre*_ (p-value < 0.01). Meanwhile, models built with Δ*F*_1_ and Δ*F*_2_ showed significantly higher AUCs than *F*_*post*1_ and *F*_*post*2_ (p-value < 0.01), respectively. These results indicate that delta-radiomics may provide better treatment assessment than single-time-point radiomics.

**Fig 5:**
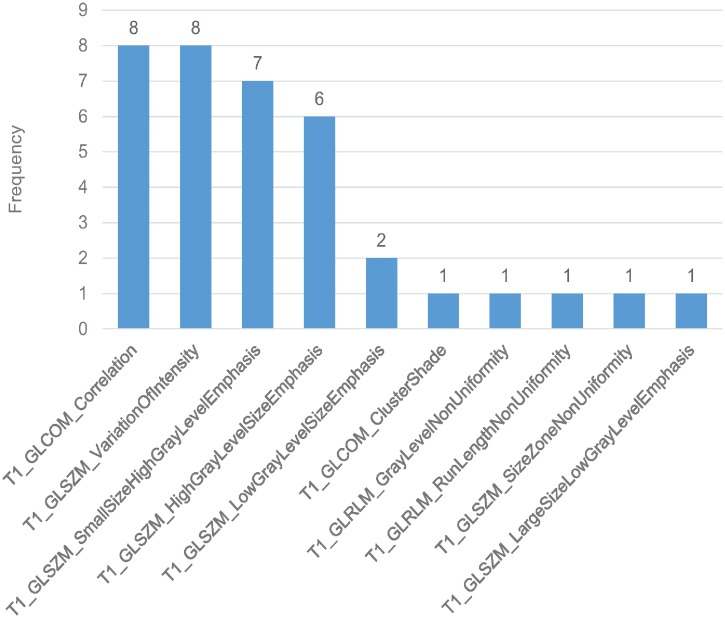
Feature selection frequency in RF + KSVM. 3 features selected in each fold of LOOCV.

**Fig 6:**
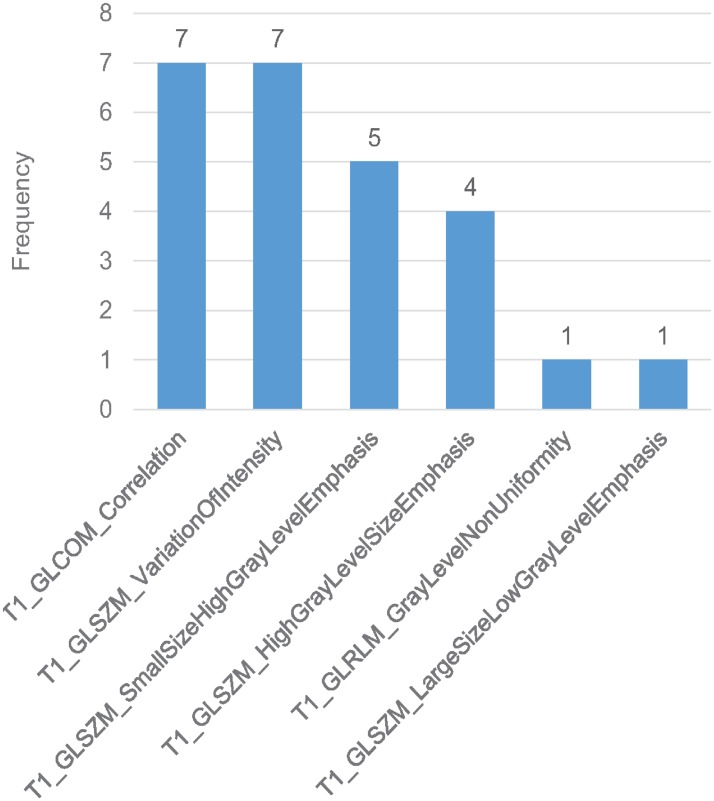
Feature selection frequency in RF + NN. 2 features selected in each fold of LOOCV.

**Fig 7:**
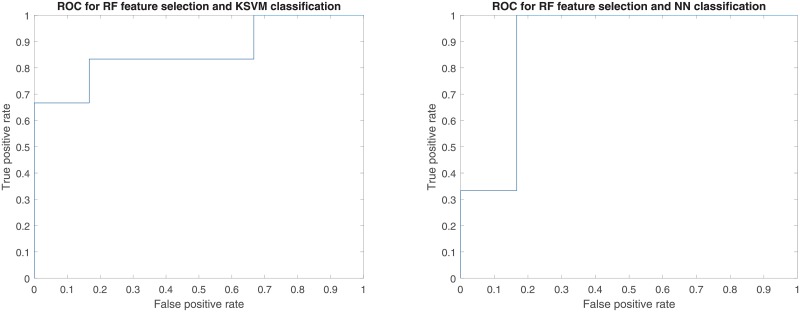
ROC curves for RF+KSVM and RF+NN combinations. Both AUC = 0.889.

As shown in Figs 3 and 4, the time point for calculating delta-radiomics and machine learning methods used affected model performance. When RF and NN were used for feature selection, ΔF_1_ resulted in higher predictive performance than ΔF_2_, independent of the type of machine learning algorithm used for classification (p-value < 0.01). When L1-LR was used for feature selection, ΔF_1_ resulted in higher AUC values than ΔF_2_ with KSVM, NN, and NB classifiers, and resulted in lower AUC values than ΔF_2_ with L1-LR, L2-LR, LSVM, and RF classifiers. The model combinations of RF+KSVM and RF+NN showed the highest AUCs (AUC = 0.889) with ΔF_1_. The number of features selected for each model combination is provided in S2 Appendix. During feature selection for two-month post-treatment features, only 2 features were selected by the univariate Cox regression model in one fold of the leave-one-out cross-validation. Therefore, only up to 2 features were tested from two-month post-treatment features. For the maximum AUC achieved, 3 features were selected in RF+KSVM combination and 2 features were selected in RF+NN combination. The frequencies of the features selected during LOOCV for these two model combinations is illustrated in Figs 5 and 6. As shown in the figures, T1 GLCOM correlation and T1 GLSZM variation of intensity are the two most frequently selected features for this cohort of patients. The ROC curves for the two model combinations are shown in Fig 7.

## IV. Discussions

This study compared single-time-point features with delta-features for the assessment of concurrent brain SRS and bevacizumab treatment outcomes using different machine learning methods. Data in Fig 3 indicated that the single-time-point features (*F*_*pre*_, *F*_*post*1_, Δ*F*_1_) and delta-features (Δ*F*_1_, Δ*F*_2_) resulted in different predictive performance for this assessment. The delta-features displayed higher predictive performance than single-time-point features. One-week delta-features resulted in better prediction than two-month delta-features for most of the tested model combinations. However, it should be noted that the results were based on a small cohort of patients. Although it is difficult to generalize the results from this small dataset, we can reasonably infer that delta-features potentially provided better predictive decision support than single-time-point features. Additionally, the time point for computing delta-features was shown to affect model performance, indicating that the time at which radiomic features are extracted may be of particular importance. These conclusions remain limited, however, further analysis on a larger dataset is required to fully validate this result.

Image acquisition and pre-processing could affect feature extraction and analysis results. In this study, the MR image quality was maintained stable by following strict quality control (QC) and quality assurance (QA) procedures. QC of the MR scanner followed 2015 ACR MRI Quality Control Manual. Daly QA was executed and key factors like gain factor were recorded. The rigid body image registration error was initially performed using an automatic registration algorithm in the Velocity software and then manually validated by checking the anatomical structures between two data sets (typically the accuracy is within 0.5mm). The GTV contours were transferred from pre-treatment images to post-treatment images. No modifications were made for GTV, because the feature change within the whole GTV region could be informative to treatment outcome. Since the same GTV was used for radiomics feature calculation, the morphological delta-features were 0 and were not selected in the feature selection process. The intensity/texture delta-features were informative by revealing the intensity/texture change within the pre-treatment GTV contour.

Few studies were found that compared single-time-point features to delta-features. A study by Zhang et. al.[23] also showed better performance of delta-features (accuracy: 73.2%) than that of features at the second time point (69.1%), which agrees with our findings. One limitation of this study is that the time separation between the two MR scans was different for each patient (9–119 days). Our study avoided this limitation by incorporating MR images at unified time differences. In the study by Fave et. al.[18], delta-radiomics were not prognostic in predicting OS or distant metastases, and only one delta-radiomics feature was prognostic in predicting local-regional recurrence. This result could be related to the limited delta-features used for building models, since delta-features were reduced when the corresponding pre-treatment features were not prognostic in their study. In our study, this limitation was minimized by applying feature selection directly on delta-features.

One limitation of this study is the limited size of the dataset. Constrained by the small sample size, selecting an appropriate number of features to build classification models is challenging. Some studies have shown that the number of features selected affected the classification model performance.[3, 4, 20, 37] From some previous experience[1, 15], 10 observations per feature have been shown to be needed to build a binary classification model using a cross-validation scheme. However, Hua et al. [38] reported the optimal number of features relies on the correlation among features, the type of classification method, and the size of dataset. For example, they reported that one can safely use a large number of features for a small dataset for linear and polynomial SVM. They also mentioned that the optimal feature number did not monotonically increase with the sample size for some classifiers like SVM and 3-nearest-neighbor. Considering both opinions and attempting to explore the effect of number of features selected for a small dataset, we included 1 to 3 features to build the classification models. During feature selection for two-month post-treatment features, only 2 features were selected by the univariate Cox regression model in one fold of the leave-one-out cross-validation. Therefore, only up to 2 features were tested from two-month post-treatment features. The variation of AUCs with the number of features selected for each feature category and each model combination was shown in S2 Appendix. The AUCs reported in Fig 3 were the highest AUCs when the optimal number of features selected for each model combination. When the same AUC was achieved by classification models built with different groups of features, the model with the smallest number of features should be selected to alleviate the overfitting problem.

The z-score normalization was used for each feature across the whole patient dataset. Conventionally, one would normalize features of training dataset to z-score distribution and then shift the features of validation dataset to share the mean and standard deviation of the training set. [39] This method is built based on the assumptions that the training dataset and validation dataset share the same distribution and the training dataset is large enough to describe the distribution. However, for a small dataset, the mean and standard deviation of z-distribution are easily influenced by a few extreme values. Normalizing the whole dataset would be better to guarantee this assumption in case the appearance of few outliers.[3, 6],^22^

To mitigate the limitation of dataset, we used leave-one-out cross-validation (LOOCV) and a two-step feature selection process. Cross-validation has been shown as an efficient method to provide an unbiased estimation on survival prediction.[40] Within each individual validation of LOOCV, the features were first selected by univariate Cox regression and then by multivariate machine learning methods. The univariate Cox regression model initially selected features which could be predictive to OS to reduce the overfitting of machine learning feature selection model. The number of features selected by univariate Cox regression model ranged from 11 to 58 during LOOCV. To avoid overfitting of classification models, the number of features selected was further reduced by multivariate machine learning feature selection models. The univariate Cox regression model did not consider the combined effect of two or more features, but this deficiency was offset by the following multivariate machine learning feature selection which considered both the relevancy between features and overall survival as well as the combined effect among the features. The AUCs calculated using the univariate Cox regression model for feature selection and using the two-step process for feature selection for delta-features are displayed in Table 1. When the two-step feature selection process was used, the AUCs in Table 1 are the highest AUCs resulted from the 3 feature selection models. As we can see from Table 1, the AUCs were overall improved with machine learning feature selection.

**Table 1:** Comparison of AUCs with/without machine learning feature selection for delta-features.

AUC	Feature selection: Cox		Feature selection: Cox + Machine Learning	
Classification	ΔF_1_	ΔF_2_	ΔF_1_	ΔF_2_
**L1-LR**	0.806	0.556	0.806	0.833
**L2-LR**	0.778	0.583	0.778	0.833
**LSVM**	0.887	0.112	0.861	0.750
**KSVM**	0.698	0.230	0.889	0.750
**RF**	0.759	0.540	0.833	0.834
**NN**	0.607	0.531	0.889	0.556
**NB**	0.264	0.292	0.819	0.528

In this study, ROC analysis was used to evaluate the performance of each model combination. Conventionally, ROC curves are generated when the decision threshold varies for models with fixed weights. In this study, the AUCs were used to compare the predictive performances of different model combinations.[41, 42] Therefore, ROC analysis was reasonably used, although the weights in machine learning models adapted to different feature values during LOOCV.

This study emphasized on the methodology for feature extraction (delta-radiomics) and feature analysis (machine learning feature selection and classification). However, image acquisition and segmentation are also important and have substantial impacts on the performance of radiomics studies. For example, variations in image acquisition and reconstruction parameters could introduce non-biological changes into the images.^1^ For image segmentation, many ROIs have indistinct borders, and there are controversies on the ground truth and reproducibility of image segmentations. In this study, the GTV contours of post-treatment images were registered from those of pre-treatment images, which may have an impact on the final decision. Those should be thoroughly investigated in the future studies while planning for comprehensive clinical trials.

## V. Conclusions

This study compared single-time-point features (pre-treatment features, one-week post-treatment features, and two-month post-treatment features) and delta-features (one-week delta-features and two-month delta-features) in assessing brain tumor radiosurgery with machine learning approaches.

The analysis results from the limited dataset implied that delta-features provide higher predictive performance than single-time-point features for this cohort of patients and may potentially be more valuable for treatment assessment than single-time-point features. The time point of computing the delta-features may also be a significant factor for the model performance. With a univariate Cox regression model as the first step for feature selection, the model combinations of RF feature selection with KSVM classification and RF feature selection with NN classification provided the highest AUCs with one-week delta-features. A larger dataset to validate the results of this study is highly desirable.

## Supporting information

S1 Appendix Features calculated in the present study. (DOCX)

S2 Appendix AUCs with varying number of features selected. (DOCX)
