# Supplementary material for: An investigation of machine learning methods in delta-radiomics feature analysis
Source: PLoS One. 2019 Dec 13;14(12):e0226348. doi: 10.1371/journal.pone.0226348 (PMC6910670; doi:10.1371/journal.pone.0226348)
Supplement: S1 Appendix — (DOCX) [file pone.0226348.s001.docx]

**S1 Appendix. Features Calculated in the Present Study**

| **Intensity histogram-based features (4)** |
| --- |
| Energy, Entropy, Kurtosis, Skewness |
| **GLCOM texture features (22)** |
| Auto Correlation, Cluster Prominence, Cluster Shade, Cluster Tendency, Contrast Correlation, Differential Entropy, Dissimilarity, Energy, Entropy, Homogeneity1, Homogeneity2, Info Measure Correlation1, Info Measure Correlation2, Inverse Difference Moment Normalized, Inverse Difference Normalized, Inverse Variance, Maximum Probability, Sum Average, Sum Entropy, Sum Variance, Variance |
| **GLRLM texture features (11)** |
| Short Run Emphasis, Long Run Emphasis, Gray Level Non-uniformity, Run Length Non-uniformity, Run Percentage, Low Gray Level Run Emphasis, High Gray Level Run Emphasis, Short Run Low Gray Level Emphasis, Short Run High Gray Level Emphasis, Long Run Low Gray Level Emphasis, Long Run High Gray Level Emphasis |
| **GLSZM texture features (13)** |
| Small Zone Emphasis, Large Zone Emphasis, Gray Level Non-uniformity, Size Zone Non-uniformity, Size Percentage, Low Gray Level Size Emphasis, High Gray Level Size Emphasis, Small Size Low Gray Level Emphasis, Small Size High Gray Level Emphasis, Large Size Low Gray Level Emphasis, Large Size High Gray Level Emphasis, Variation Of Intensity, Variation Of Area |
| **NGLDM texture features (5)** |
| Coarseness, Contrast, Busyness, Complexity, Texture Strength |
| **Morphological features (6)** |
| Compactness1, Compactness2, Sphericity, Spherical Disproportion, Surface Area, Volume |
