# Supplementary material for: An investigation of machine learning methods in delta-radiomics feature analysis
Source: PLoS One. 2019 Dec 13;14(12):e0226348. doi: 10.1371/journal.pone.0226348 (PMC6910670; doi:10.1371/journal.pone.0226348)
Supplement: S2 Appendix — (DOCX) [file pone.0226348.s002.docx]

**S2 Appendix. AUCs with Varying Number of Features Selected**

Table 1. Variation of AUC with number of features selected by L1-LR

| Feature category | Classification method | Number of features selected | | |
| --- | --- | --- | --- | --- |
|  |  | 1 | 2 | 3 |
| $\boldsymbol{F}_{\boldsymbol{pre}}$ | L1-LR | 0.306 | 0.250 | 0.222 |
|  | L2-LR | 0.306 | 0.250 | 0.222 |
|  | LSVM | 0.167 | 0.222 | 0.250 |
|  | KSVM | 0.222 | 0.278 | 0.194 |
|  | RF | 0.149 | 0.199 | 0.239 |
|  | NN | 0.430 | 0.491 | 0.442 |
|  | NB | 0.194 | 0.194 | 0.167 |
| $\mathbf{F}_{\mathbf{post1}}$ | L1-LR | 0.472 | 0.583 | 0.722 |
|  | L2-LR | 0.472 | 0.583 | 0.722 |
|  | LSVM | 0.361 | 0.639 | 0.667 |
|  | KSVM | 0.194 | 0.500 | 0.667 |
|  | RF | 0.286 | 0.488 | 0.609 |
|  | NN | 0.476 | 0.534 | 0.547 |
|  | NB | 0.111 | 0.333 | 0.444 |
| $\mathbf{F}_{\mathbf{post2}}$ | L1-LR | 0.611 | 0.611 | N.A. |
|  | L2-LR | 0.611 | 0.611 | N.A. |
|  | LSVM | 0.472 | 0.500 | N.A. |
|  | KSVM | 0.361 | 0.556 | N.A. |
|  | RF | 0.314 | 0.439 | N.A. |
|  | NN | 0.523 | 0.515 | N.A. |
|  | NB | 0.417 | 0.278 | N.A. |
| $\Delta\boldsymbol{F}_{\mathbf{1}}$ | L1-LR | 0.667 | 0.694 | 0.667 |
|  | L2-LR | 0.667 | 0.694 | 0.667 |
|  | LSVM | 0.611 | 0.667 | 0.694 |
|  | KSVM | 0.722 | 0.833 | 0.694 |
|  | RF | 0.773 | 0.756 | 0.694 |
|  | NN | 0.539 | 0.603 | 0.694 |
|  | NB | 0.444 | 0.472 | 0.694 |
| $\Delta\boldsymbol{F}_{\mathbf{2}}$ | L1-LR | 0.722 | 0.806 | 0.833 |
|  | L2-LR | 0.722 | 0.806 | 0.833 |
|  | LSVM | 0.528 | 0.750 | 0.750 |
|  | KSVM | 0.306 | 0.389 | 0.750 |
|  | RF | 0.766 | 0.833 | 0.834 |
|  | NN | 0.534 | 0.522 | 0.539 |
|  | NB | 0.347 | 0.333 | 0.361 |

Table 2. Variation of AUCs with number of features selected by RF

| Feature Category | Classification Method | Number of features selected | | |
| --- | --- | --- | --- | --- |
|  |  | 1 | 2 | 3 |
| $\boldsymbol{F}_{\boldsymbol{pre}}$ | L1-LR | 0.278 | 0.306 | 0.306 |
|  | L2-LR | 0.278 | 0.306 | 0.306 |
|  | LSVM | 0.222 | 0.250 | 0.194 |
|  | KSVM | 0.500 | 0.417 | 0.361 |
|  | RF | 0.278 | 0.250 | 0.278 |
|  | NN | 0.472 | 0.250 | 0.278 |
|  | NB | 0.222 | 0.222 | 0.194 |
| $\mathbf{F}_{\mathbf{post1}}$ | L1-LR | 0.639 | 0.694 | 0.694 |
|  | L2-LR | 0.639 | 0.694 | 0.694 |
|  | LSVM | 0.694 | 0.667 | 0.639 |
|  | KSVM | 0.694 | 0.722 | 0.722 |
|  | RF | 0.694 | 0.625 | 0.431 |
|  | NN | 0.528 | 0.806 | 0.528 |
|  | NB | 0.681 | 0.569 | 0.667 |
| $\mathbf{F}_{\mathbf{post2}}$ | L1-LR | 0.389 | 0.472 | N.A. |
|  | L2-LR | 0.389 | 0.472 | N.A. |
|  | LSVM | 0.194 | 0.361 | N.A. |
|  | KSVM | 0.167 | 0.472 | N.A. |
|  | RF | 0.153 | 0.264 | N.A. |
|  | NN | 0.389 | 0.444 | N.A. |
|  | NB | 0.194 | 0.111 | N.A. |
| $\Delta\boldsymbol{F}_{\mathbf{1}}$ | L1-LR | 0.722 | 0.778 | 0.806 |
|  | L2-LR | 0.722 | 0.750 | 0.778 |
|  | LSVM | 0.722 | 0.861 | 0.861 |
|  | KSVM | 0.639 | 0.861 | 0.889 |
|  | RF | 0.542 | 0.556 | 0.833 |
|  | NN | 0.694 | 0.889 | 0.417 |
|  | NB | 0.583 | 0.819 | 0.819 |
| $\Delta\boldsymbol{F}_{\mathbf{2}}$ | L1-LR | 0.667 | 0.722 | 0.750 |
|  | L2-LR | 0.667 | 0.722 | 0.750 |
|  | LSVM | 0.556 | 0.639 | 0.667 |
|  | KSVM | 0.250 | 0.306 | 0.722 |
|  | RF | 0.722 | 0.750 | 0.653 |
|  | NN | 0.278 | 0.556 | 0.361 |
|  | NB | 0.319 | 0.458 | 0.528 |

Table 3. Variation of AUCs with number of features selected by NN

| Feature Category | Classification Method | Number of features selected | | |
| --- | --- | --- | --- | --- |
|  |  | 1 | 2 | 3 |
| $\boldsymbol{F}_{\boldsymbol{pre}}$ | L1-LR | 0.428 | 0.389 | 0.387 |
|  | L2-LR | 0.428 | 0.389 | 0.383 |
|  | LSVM | 0.212 | 0.193 | 0.253 |
|  | KSVM | 0.294 | 0.331 | 0.325 |
|  | RF | 0.314 | 0.277 | 0.301 |
|  | NN | 0.453 | 0.506 | 0.442 |
|  | NB | 0.329 | 0.304 | 0.305 |
| $\mathbf{F}_{\mathbf{post1}}$ | L1-LR | 0.621 | 0.632 | 0.713 |
|  | L2-LR | 0.621 | 0.632 | 0.712 |
|  | LSVM | 0.507 | 0.553 | 0.616 |
|  | KSVM | 0.450 | 0.452 | 0.477 |
|  | RF | 0.558 | 0.547 | 0.525 |
|  | NN | 0.542 | 0.497 | 0.497 |
|  | NB | 0.403 | 0.419 | 0.418 |
| $\mathbf{F}_{\mathbf{post2}}$ | L1-LR | 0.513 | 0.509 | N.A. |
|  | L2-LR | 0.513 | 0.507 | N.A. |
|  | LSVM | 0.399 | 0.409 | N.A. |
|  | KSVM | 0.366 | 0.395 | N.A. |
|  | RF | 0.403 | 0.382 | N.A. |
|  | NN | 0.473 | 0.538 | N.A. |
|  | NB | 0.343 | 0.327 | N.A. |
| $\Delta\boldsymbol{F}_{\mathbf{1}}$ | L1-LR | 0.694 | 0.735 | 0.775 |
|  | L2-LR | 0.694 | 0.734 | 0.769 |
|  | LSVM | 0.626 | 0.706 | 0.744 |
|  | KSVM | 0.577 | 0.701 | 0.741 |
|  | RF | 0.593 | 0.642 | 0.702 |
|  | NN | 0.537 | 0.542 | 0.604 |
|  | NB | 0.555 | 0.596 | 0.616 |
| $\Delta\boldsymbol{F}_{\mathbf{2}}$ | L1-LR | 0.537 | 0.549 | 0.574 |
|  | L2-LR | 0.539 | 0.549 | 0.574 |
|  | LSVM | 0.439 | 0.421 | 0.379 |
|  | KSVM | 0.106 | 0.123 | 0.218 |
|  | RF | 0.381 | 0.398 | 0.397 |
|  | NN | 0.509 | 0.508 | 0.488 |
|  | NB | 0.314 | 0.295 | 0.299 |
